# Oxidative stress controls lncRNA-mediated sow granulosa cell functions in a FoxO1-dependent manner

**DOI:** 10.1186/s40104-024-01120-6

**Published:** 2024-12-16

**Authors:** Wenmin Sheng, Miaomiao Wang, Yuqi Li, Zhenyu Sun, Xing Du, Qifa Li

**Affiliations:** College of Animal Science and Technology, Nanjing Agricultural University, Nanjing, 210095 China

**Keywords:** FoxO1, Granulosa cell apoptosis, *NORSF*, Oxidative stress, Sow fertility

## Abstract

**Background:**

Oxidative stress (OS) is involved in low female fertility by altering multi-omics such as the transcriptome, miRome, and lncRNome in follicular cells and follicular fluid. However, the mechanism by which OS affects multi-omics dynamics remains largely unknown. Here, we report that OS induces lncRNome dynamics in sow granulosa cells (sGCs), which is partially dependent on the transcription factor activity of its effector, FoxO1.

**Results:**

A total of 2,283 putative FoxO recognition elements (FREs) were identified in the promoters of 394 lncRNAs, accounting for 91.20% (394/432) of the lncRNAs regulated by OS. ChIP and reporter assays showed that the effector FoxO1 mediated OS regulation of lncRNA transcription in a transcription factor activity-dependent manner. In sGCs, OS induces the transcription and function (e.g., apoptosis) of *NORSF* (non-coding RNA involved in sow fertility), a nuclear lncRNA involved in sGC function via FoxO1. Furthermore, FoxO1 has been identified as a transcriptional activator of *NORSF* in sGCs that interacts with the FRE motif of its promoter. Meanwhile, OS downregulates the transcription of *CYP19A1*, which encodes an essential enzyme for estrogen synthesis and 17β-estradiol (E2) release by sGCs via the FoxO1 and *NORSF* axis. Phenotypically, dysregulation of *NORSF* transcription caused by 2 novel adjacent transitions in the promoter leads to decreased sow fertility.

**Conclusion:**

These results suggest a model of OS-stimulated lncRNome dynamics in sGCs and a new signaling pathway of OS that influences sGC function and sow fertility.

**Supplementary Information:**

The online version contains supplementary material available at 10.1186/s40104-024-01120-6.

## Introduction

Gene-environment interactions (GEI) determine all phenotypes of organisms, including sow reproductive traits, and their levels determine the economic benefits for the swine industry. Oxidative stress (OS) is a common mechanism underlying various internal and external environmental factors (e.g., heat stress, density, social rank, and feed mycotoxins) that result in low fertility [1–3]. Measures to reduce OS, such as adding antioxidant stress additives to the feed or reducing density, can improve sow fertility [1, 3–5]. OS leads to various abnormalities (e.g., apoptosis, autophagy, meiotic arrest, and oxidative damage) and multiple omics disorders in follicular cells, including sow granulosa cells (sGCs), oocytes, luteal cells of sow ovaries, while antioxidants can reverse these processes [6–9]. A more recent report showed that OS affects sGC functions and sow fertility through miR-23a, the first causal microRNA (miRNA) for sow fertility traits [10].

Interestingly, OS has also been shown to control sow ovarian function by interacting with non-coding RNAs (ncRNAs), including miRNAs and long ncRNAs (lncRNAs) [7, 11, 12]. Among the miRNAs, 55 were found to participate in the stimulation of OS on sGC apoptosis [7]. OS induces sGC apoptosis and oxidative damage via the miR-192 and *Acvr2a* axis [11]. On the other hand, several miRNAs (e.g., miR-183 cluster and let-7a) rescue OS-induced sGC apoptosis by interacting with FoxO1, a well-known sensor and effector of OS [13] and an apoptosis-inducing factor [14]. Among lncRNAs, *SDNOR* restrained by OS suppresses OS-induced sGC apoptosis and maintains the state and function of sGCs [12]. Additionally, *NORHA*, a lncRNA upregulated during sow follicular atresia, can synergistically induce FoxO1 expression with OS, thereby increasing the rate of sGC apoptosis [13]. Overall, little is known about the interactions between OS and ncRNAs in the regulation of sow ovarian function.

We previously demonstrated that OS can cause significant changes in lncRNAome dynamics in sGCs, and identified hundreds of differentially expressed lncRNAs (DElncRNAs) [12]. However, the mechanism by which OS influences the transcription of these DElncRNAs is still unclear. The effector of OS, FoxO1, is also a transcription factor (TF) that activates or inhibits target genes by interacting with the FoxO1 response elements (FREs) in their promoters [15, 16]. In this study, we aimed to explore the mechanism by which OS regulates DElncRNA transcription from the perspective of the TF activity of its effector FoxO1.

## Materials and methods

### Materials

The following reagents and kits were used: TRIzol (#RG-51001 A; Angle Gene, Nanjing, China), H_2_O_2_ (#MFCD00011333; Sinopharm, Shanghai, China), Lipofectamine 2000 (#11668500; Thermo Fisher Scientific, Waltham, MA, USA), trypsin (#25200072; Thermo Fisher Scientific), HiScript II Q Select RT SuperMix (#R232-01; Vazyme, Nanjing, China) for reverse transcription, AceQ qPCR SYBR Green Master Mix (#Q111-02; Vazyme), a DLR™ assay system for luciferase assay (#E1910; Promega, Madison, WI, USA) and a Detection Kit for Estrodiol (E2) (#E9096-E2; North Biotech, Beijing, China). DMEM and RPMI 1640 medium were ordered from Gibco (#C11330500BT; Carlsbad, CA, USA) and Hyclone (#SH30027.01; Logan, UT, USA). The anti-FoxO1 (1:1,000; #D194961) and anti-IgG (1:1,000; #D111024) antibodies were purchased from Sangon (Shanghai, China). The KGN cell line was obtained from Yili (#DCX-1125; Shanghai, China).

### Sows

In total, 200 fresh ovaries were isolated from 100 newly slaughtered commercial sows (180 days old, weighing approximately 110 kg) that were healthy and sexually mature (Zhushun, Nanjing, China). The ear tissue blocks were collected from 310 healthy Yorkshire sows with fertility records (Kangle, Jintan, China).

### Bioinformatics

The promoter sequences were downloaded from the NCBI database (https://www.ncbi.nlm.nih.gov/), and the TF-binding sites (TFBSs) were predicted using the JASPAR database (http://jaspar.genereg.net/). DAVID bioinformatics (https://david.ncifcrf.gov/) was used for Gene Ontology (GO) and Kyoto Encyclopedia of Genes and Genomes (KEGG) analyses. Data on tissue expression and quantitative trait loci (QTLs) were obtained from the NCBI and Animal QTLdb databases (https://www.animalgenome.org/), respectively. DNA–RNA triplexes in the FoxO1 promoter were detected using the Gaemons software (http://www.gaemons.net/LongTarget).

### Constructs and small interfering RNAs (siRNAs)

Overexpression plasmids for *NORSF* (non-coding RNA involved in sow fertility) and *FoxO1* have been previously constructed [13, 17]. Promoters harboring wild-type or mutated versions of the TFBSs were ligated into the pGL3-basic vector (#E1751, Promega), and the primers are given in Table S1. *FoxO1*- and *NORSF*-specific siRNAs (Table S2) were previously validated by our group [13, 17], and synthesized with Generay (Shanghai, China).

### Cells and treatment

Fresh ovaries were cleaned three times with saline at 37 °C and 75% alcohol supplemented with gentamicin. Antral follicles of 3–5 mm were isolated, and the follicular fluid was suctioned using a UV-sterilized syringe and centrifuged at 1,000 r/min for 5 min. After discarding the supernatant, the cells were resuspended in PBS and centrifuged to collect sGCs. sGCs were cultured in DMEM with 15% FBS. KGN cells were cultured in RPMI 1640 with 15% FBS. After culture for 12 h, constructs and siRNAs were transfected with Lipofectamine 2000 according to the manufacturer’s instructions. After transfection for 12 h, cells were resuspended in DMEM without FBS, and treated with H_2_O_2_ at a final concentration of 150 µmol/L and for 2 h.

### Reporter assays

KGN cells were co-treated with expression plasmids, reporter constructs, and pRL-TK for 24 h, and then collected for lysis and measurement. Luciferase activity for each sample was obtained using an enzyme-labeling instrument (Thermo Fisher Scientific) and normalized to the pRL-TK levels.

### Chromatin immunoprecipitation (ChIP)

ChIP was conducted as described previously [10]. After crosslinking in formaldehyde, precipitating chromatin by centrifugation, and sonication, the samples were precleared with protein A/G agarose beads and incubated with anti-FoxO1 antibody. Primers (Table S3) and qPCR were used to amplify the input and bound DNA. The negative control was DNA enriched with the IgG antibody, and the input control was unprocessed DNA.

### Quantitative real-time PCR (qPCR)

sGCs were collected 24 h post-transfection to extract total RNA using TRIzol reagent. Reverse transcription and qPCR were conducted with the corresponding kits and procedures [10]. The 2^−ΔΔCT^ method was employed to calculate the transcription level, with the *GAPDH* and *RPLP0* serving as the reference controls. The primers are given in Table S4.

### Fluorescence-activated cell sorting (FACS)

After washing twice with pre-cooled PBS, sGCs were dyed with 5 µL of each Annexin V and Propidium Iodide (PI) for 10 min. The sGCs were then diluted with Binding Buffer and sorted by flow cytometry (Becton Dickinson, Franklin Lakes, NJ, USA). The sGCs count in each quadrant was read using FlowJo v7.6 software (TreeStar, Ashland, OR, USA).

### SNV screening, genotyping, and association analysis

Identification of single nucleotide variations (SNVs) and genotyping were conducted using DNA sequencing. SAS v9.2 software (SAS Institute, Cary, NC, USA) was employed for association analysis with linear models in literature [18]. Fertility traits included the total number of piglets born (TNB), number of piglets born alive (NBA), number of healthy piglets (NHP), number of stillbirths (NSB), and litter weight (LW). The primers are given in Table S5.

### Statistics

Two-tailed *t*-test and one-way analysis of variance in Prism v9.0 software (GraphPad, San Diego, CA, USA) were used to evaluate the data. Each experiment was independently conducted in triplicates. The level of significance was set at *P* < 0.05.

## Results

### OS stimulates the lncRNome of sGCs in a FoxO1-dependent manner

**Fig. 1:**
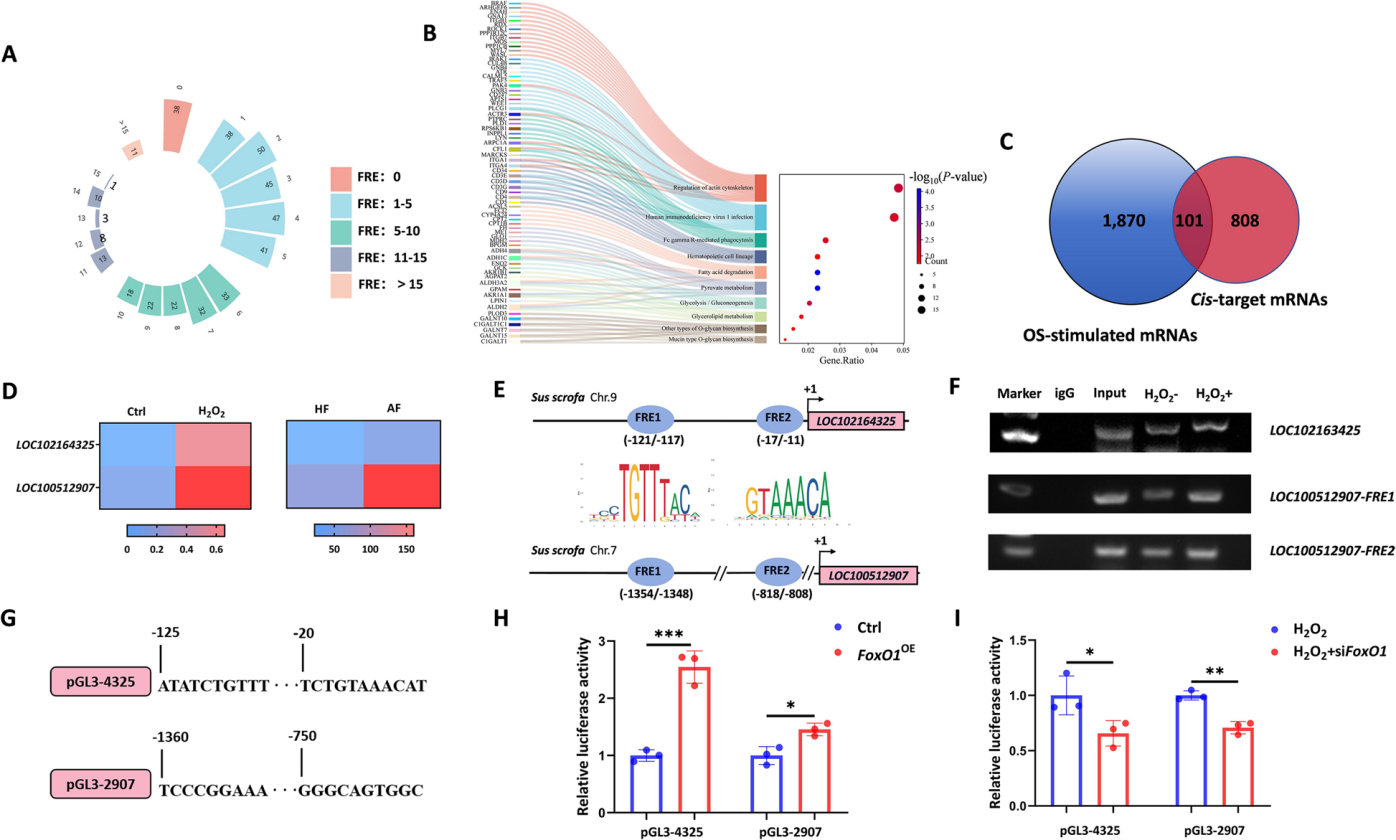
OS stimulates lncRNome in a FoxO1-dependent manner. **A** Diagram depicting the putative FRE motifs in the promoters of DElncRNAs stimulated by OS. DElncRNAs were obtained from our previous study [12]. **B** KEGG analysis of *cis*-target mRNAs of DElncRNAs was conducted with DAVID bioinformatics (https://david.ncifcrf.gov/). **C** Diagram depicting *cis*-target mRNAs that are differentially expressed in OS-stimulated sGCs. Data for mRNAs in OS-stimulated sGCs were obtained from our previous report [7]. **D** Two DElncRNAs in both OS-stimulated sGCs and follicular atresia process. DElncRNAs in the follicular atresia process were obtained from our previous study [17]. **E** Diagram depicting the FRE motifs in the promoters of 2 DElncRNAs. **F** ChIP assays. ChIP-PCR was conducted with an anti-FoxO1 antibody in sGCs, and detected with agarose gel electrophoresis. **G**–**I** Reporter assays. The reporter constructs of promoter harboring FRE motifs (**G**) were transfected into sGCs, luciferase activities were measured (**H**), or re-treated with H_2_O_2_ and *FoxO1*-siRNA, luciferase activities were measured (**I**). Quantitative data are plotted as mean ± standard error. ^*^P < 0.05. ^**^P < 0.01

To understand whether FoxO1 participates as a TF in the OS-stimulated lncRNome dynamics of sGCs, we predicted FRE motifs in the promoters of 432 OS-stimulated DElncRNAs [12]. In total, 2,283 putative FRE motifs were identified, and 394 DElncRNAs were detected with at least one FRE motif, accounting for 91.20% (394/432) of the DElncRNAs stimulated by OS (Fig. 1A and Table S6). GO and KEGG analyses showed that 909 potential *cis*-target mRNAs of the 394 DElncRNAs were markedly enriched in pyruvate metabolism, fatty acid degradation, and regulation of actin cytoskeleton (Fig. 1B, S1, Tables S7 and S8). Additionally, 101 potential *cis*-target mRNAs were differentially expressed in OS-stimulated sGCs, with 24.62% (97/394) of DElncRNAs having at least one *cis*-target mRNAs regulated by OS (Fig. 1C and Table S9). Together, these data suggest that OS stimulates the lncRNome of sGCs, which may be related to the TF activity of its effector FoxO1.

OS and its effector FoxO1 have been shown to be related to the sow follicular atresia [10, 13]. Therefore, we conducted a joint analysis of the lncRNAome of sGCs stimulated by OS and the lncRNAome of sow follicular atresia [17] and found that of 19 DElncRNAs during follicular atresia, 2 (*LOC100512907* and *LOC102164325*) were stimulated by OS (Fig. 1D). Interestingly, the promoters of these 2 DElncRNAs contained 2 FRE motifs each (Fig. 1E). Next, we investigated whether FoxO1 mediated the regulation of target lncRNAs in sGCs in response to OS by directly interacting with their promoters. As expected, FoxO1 physically interacted with the promoters of these 2 DElncRNAs, and this interaction was stimulated by OS (Fig. 1F). Furthermore, reporter assays showed that FoxO1 increased the promoter activities of these 2 DElncRNAs (Fig. 1G–H). Additionally, depleting FoxO1 restrained the increase in the promoter activities of these two DElncRNAs stimulated by OS (Fig. 1I), indicating that FoxO1 can mediate regulation of the promoter activities of these 2 DElncRNAs in response to OS. Taken together, our results support that OS controls the transcriptional activity of lncRNAs in sGCs in a FoxO1-dependent manner.

### FoxO1 mediates OS induction of *NORSF* transcription and cell apoptosis

**Fig. 2:**
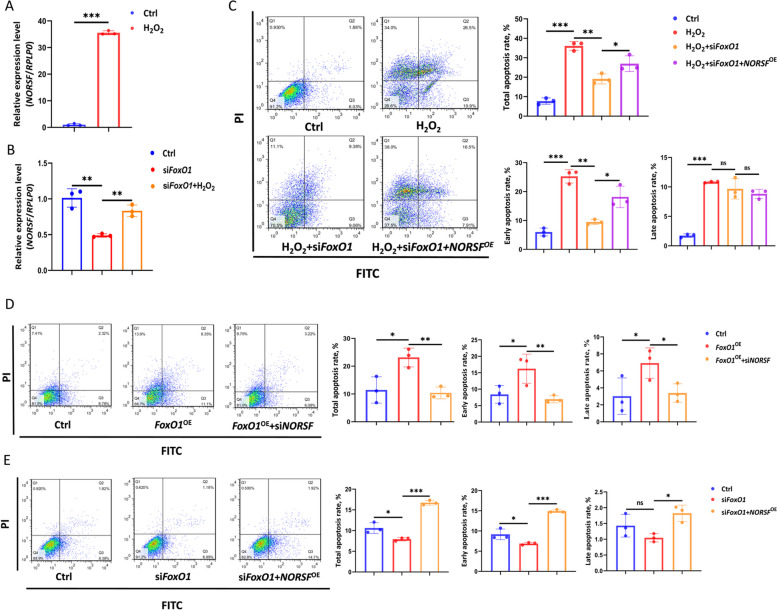
FoxO1 mediates OS induction of *NORSF* transcription and its role in sGC apoptosis. **A** *NORSF* transcription levels were determined in sGCs treated with H_2_O_2_. **B** *NORSF* transcription levels were determined in sGCs co-treated with H_2_O_2_ and *FoxO1*-siRNA. **C** Apoptosis rate was determined in sGCs co-treated with H_2_O_2_, *FoxO1*-siRNA, and pcDNA3.1-*NORSF. ***D** and **E** *FoxO1*-siRNA and pcDNA3.1-*NORSF* (**D**) or pcDNA3.1-*FoxO1* and *NORSF*-siRNA (**E**) were co-transfected into sGCs, and the apoptosis rate was determined. Quantitative data are plotted as mean ± standard error. ^*^P < 0.05. ^**^P < 0.01. ^***^P < 0.001. ns, no significance

We noticed that *LOC102164325* (also known as *NORSF*) is intensely involved in sow follicular atresia [17]. Thus, *NORSF* was selected as the target to analyze FoxO1 mediated role of OS in subsequent studies. As expected, a marked increase in *NORSF* transcription levels was observed in sGCs under OS triggered by H_2_O_2_ exposure (Fig. 2A). Furthermore, depleting *FoxO1* in sGCs resulted in a remarkable decrease in *NORSF* transcription levels, whereas OS triggered by H_2_O_2_ exposure reversed this process (Fig. 2B). The above results suggest that the effector FoxO1 mediates the OS induction of lncRNA *NORSF* transcription in sGCs.

OS is an essential factor for inducing apoptosis in sGCs [10, 13]. We first confirmed that OS triggered by H_2_O_2_ exposure induced sGC apoptosis (Fig. 2C). However, this process was reversed by depleting *FoxO1* (Fig. 2C), confirming that OS induces apoptosis via its effector FoxO1 in sGCs. Similarly, depleting *FoxO1* in sGCs resulted in a remarkable decrease in the apoptosis rate (Fig. 2E). Simultaneously, overexpression of *NORSF* partially increased the rate of apoptosis (Fig. 2D). The same results were observed for OS triggered by H_2_O_2_ exposure (Fig. 2C). Additionally, depleting *NORSF* transcript restrained the decrease in the apoptosis rate caused by the overexpression of *FoxO1* (Fig. 2D). These data support the conclusion that OS and its effector, FoxO1, control sGC apoptosis through inducing *NORSF* transcription.

### FoxO1 controls *NORSF* transcriptional activity via the FRE1 motif in its core promoter

**Fig. 3:**
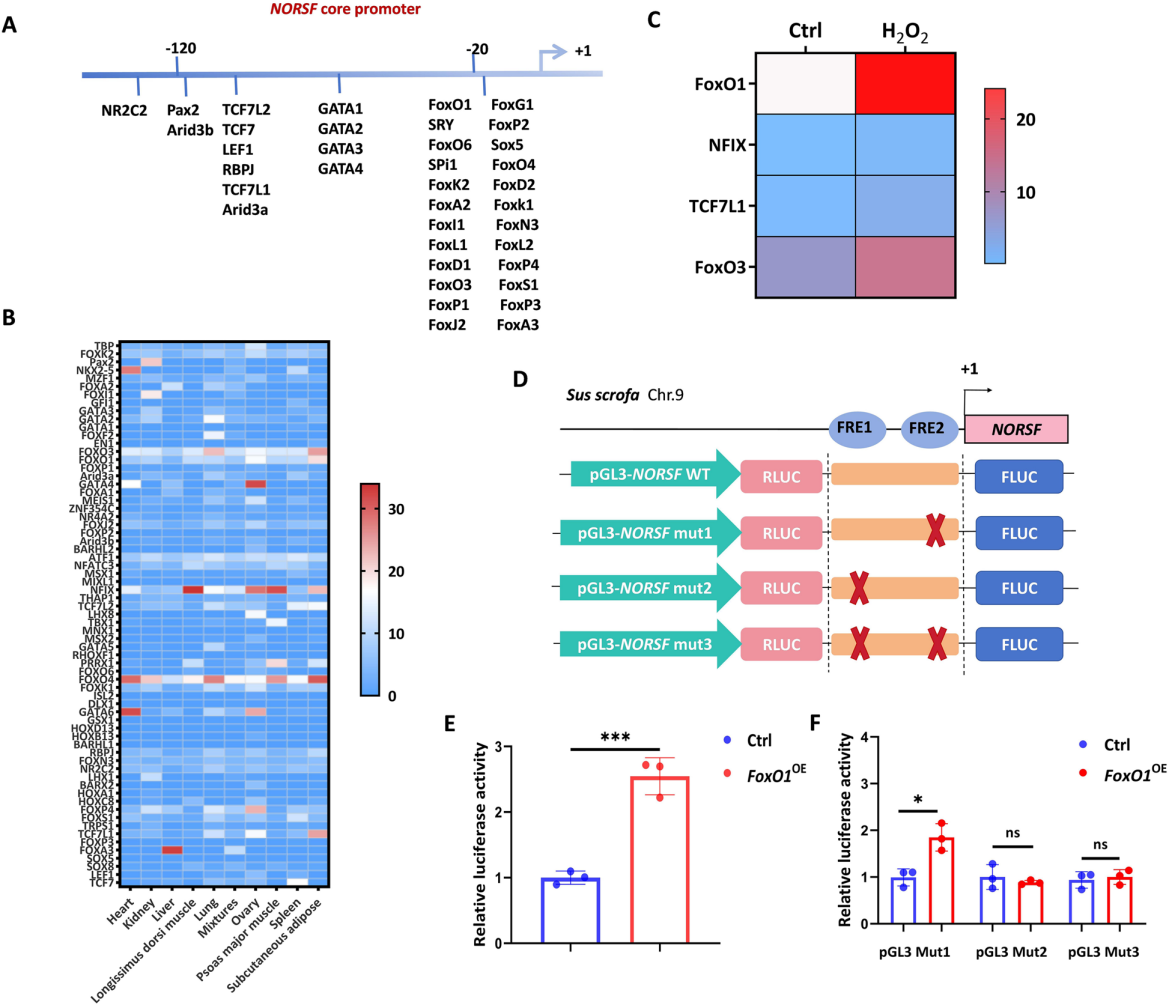
FoxO1 controls *NORSF* transcription activity via interaction with its promoter. **A** Diagram depicting the potential TFBSs in the core promoter of the *NORSF* gene. The core promoter was identified with our group and located at -139/-1 nt (unpublished), and only TFs with relative scores ≥ 0.95 were listed. **B** Electronic tissue expression profiling of TFs in swine. Data for tissue expression were obtained from the NCBI database. **C** The expression of TFs in OS-stimulated sGCs. Data for mRNAs in OS-stimulated sGCs were obtained from our previous report [7]. **D** Diagram depicting the reporter constructs of the *NORSF* core promoter. **E** and **F** Reporter assay. The reporter constructs and pcDNA3.1-*FoxO1* were co-transfected into sGCs, and luciferase activities were measured. Quantitative data are plotted as mean ± standard error. ^*^P < 0.05. ^***^P < 0.001. ns, no significance

We previously identified the *NORSF* core promoter [19]. Next, we predicted the potential TFBSs in this region and identified 149 TFBSs of 79 TFs with a relative score ≥ 0.90 (Fig. 3A; Table S10). These TFs were significantly enriched in endometrial cancer, acute myeloid leukemia, and FoxO signaling pathway (Fig. S2). Electronic tissue expression profiling showed that nine TFs, such as GATA4, FoxO1, and NFIX, were relatively abundant in sow ovarian tissue (Fig. 3B; Table S11). Additionally, four TFs, including FoxO1, NFIX, TCF7L1, and FoxO3, were observed to be stimulated by OS triggered by H_2_O_2_ exposure (Fig. 3C) using our previous transcriptome data from OS-stimulated sGCs [7]. Combined with previous ChIP assays, these data suggest that FoxO1 is a critical TF for *NORSF* in sGCs.

We have previously confirmed that the TF FoxO1 directly interacts with the promoter of *NORSF* gene to enhance its transcriptional activity (Fig. 1). Furthermore, 2 FRE motifs are located at -121/-117 nt (FRE1 motif) and -18/-11 nt (FRE2) (Fig. 3D). Therefore, we investigated whether FoxO1 induces *NORSF* transcriptional activity through specific motifs, and generated the reporter constructs of the *NORSF* core promoter harboring the FRE motifs (Fig. 3D). Compared to the empty vector, transfection of the *FoxO1* overexpression vector gave a remarkable induction in the activity of *NORSF* core promoter (Fig. 3E). Furthermore, FoxO1 induced the activity of *NORSF* promoter via the FRE1 motif in the core promoter (Fig. 3F). Combined with previous ChIP assays, our data suggest that FoxO1 induces *NORSF* transcription in sGCs by physically interacting with the FRE1 motif of *NORSF* core promoter.

### FoxO1 is a transcription activator of *NORSF* in sGCs

**Fig. 4:**
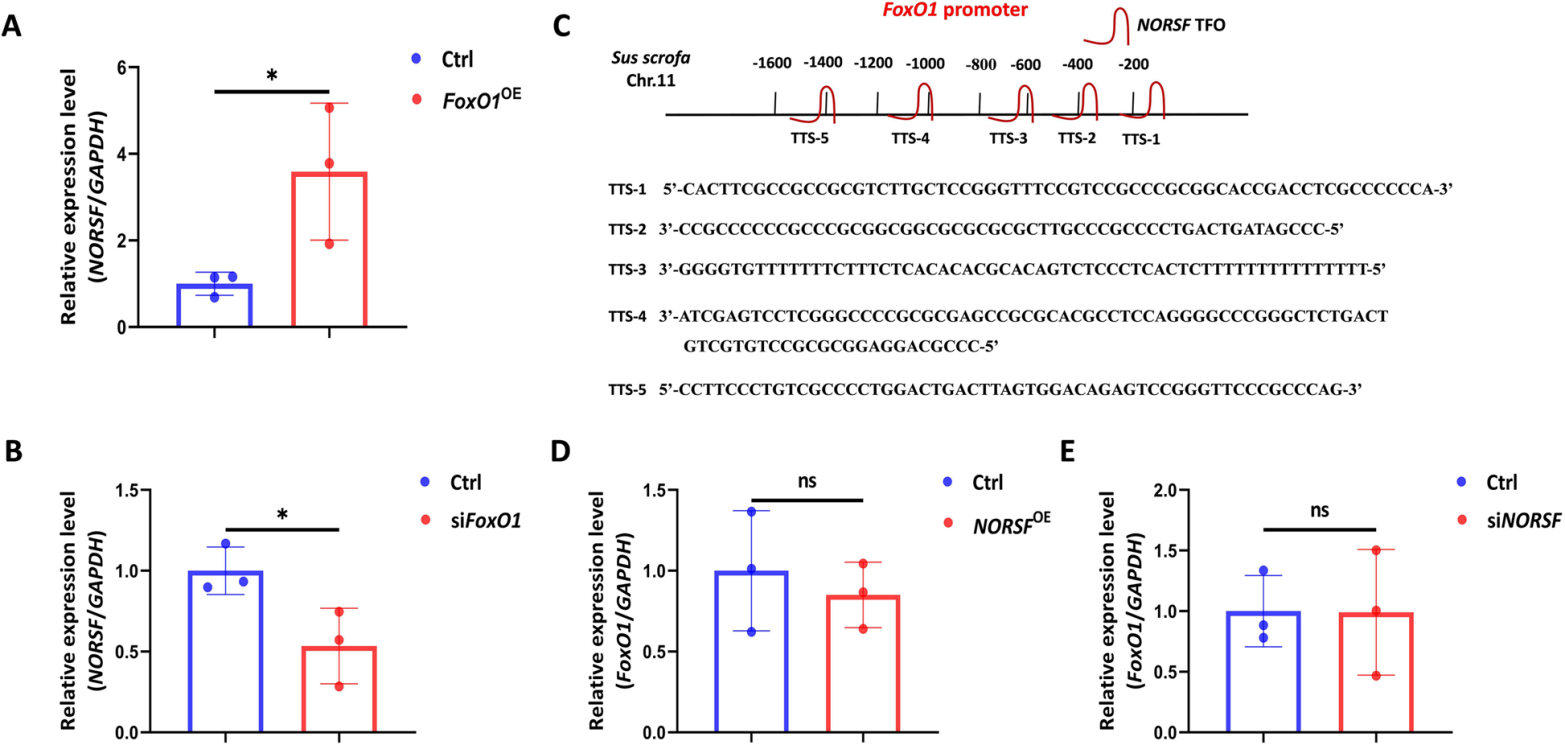
FoxO1 induces *NORSF* transcription in sGCs. **A** and **B** *NORSF* transcript levels in *FoxO1*-overexpressing (**A**) or -depleting sGCs. **C** Diagram depicting the putative DNA–RNA triplex structures formed by *NORSF* transcripts and *FoxO1* promoter. The DNA–RNA triplexes were predicted with a Gaemons. **D** and **E** *FoxO1* transcription levels in *NORSF*-overexpressing or -depleting sGCs. Quantitative data are plotted as mean ± standard error. ^*^P < 0.05. ns, no significance

Next, we investigated the effect of FoxO1 on endogenous *NORSF* transcription in sGCs. Compared to the empty vector, transfection with the *FoxO1* expression construct gave a remarkable increase in the levels of *NORSF* transcripts in sGCs (Fig. 4A). In contrast, silencing of *FoxO1* gave a noteworthy reduction in the levels of *NORSF* transcripts in sGCs (Fig. 4B). Combined with the reporter assay and ChIP results, these data support that FoxO1 is a vital transcriptional activator of the lncRNA *NORSF* in sGCs.

Our previous study showed that *NORSF* is a nuclear lncRNA in sGCs [17]. Nuclear lncRNAs usually control the transcription of targets by forming a DNA–RNA triplex with their promoters [20, 21]. Interestingly, 5 putative triplex target sites (TTSs) of the *NORSF* transcript were detected in the porcine *FoxO1* promoter and five putative triplex-forming oligonucleotides (TFOs) were detected in the *NORSF* transcript (Fig. 4C), suggesting that nuclear *NORSF* may potentially bind to the *FoxO1* promoter. However, we did not observe significant changes in *FoxO1* mRNA levels in sGCs overexpressing *NORSF* transcript (Fig. 4D and E), indicating that *NORSF* has no feedback regulatory effect on the transcription activator *FoxO1* transcription in sGCs.

### The FoxO1 and *NORSF* axis mediates OS reduction of E2 release

**Fig. 5:**
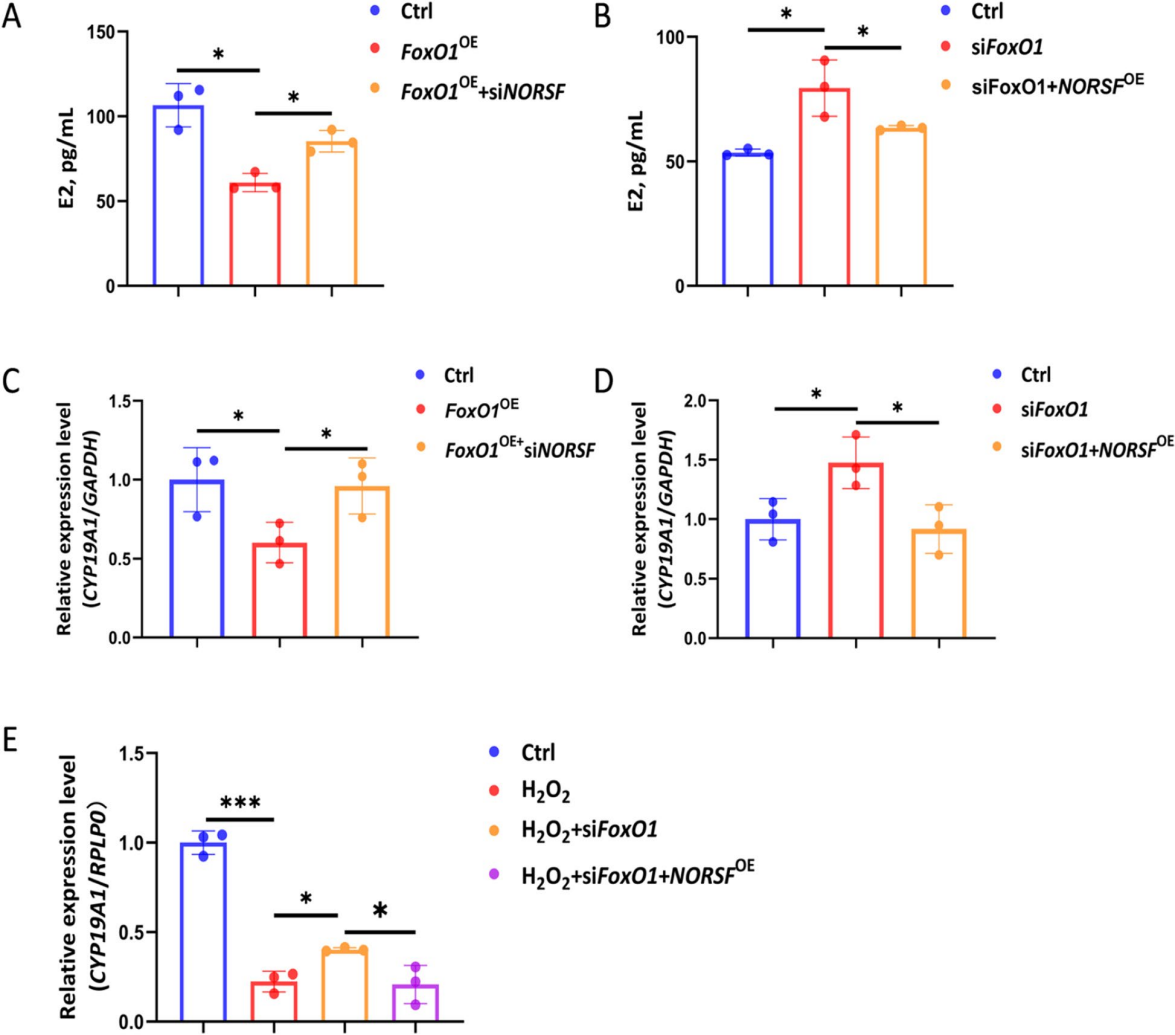
The FoxO1 and *NORSF* axis mediates OS reduction of E2 release by sGCs. **A** and **B** E2 concentration in the culture medium of sGCs co-transfecting with pcDNA3.1-*FoxO1* and *NORSF*-siRNA (**A**) or *FoxO1*-siRNA and pcDNA3.1-*NORSF* (**B**). **C** and **D** *CYP19A1* transcription levels in sGCs co-transfecting with pcDNA3.1-*FoxO1* and *NORSF*-siRNA (**C**) or *FoxO1*-siRNA and pcDNA3.1-*NORSF* (**D**). **E** *CYP19A1* transcription levels in sGCs co-treating with H_2_O_2_, *FoxO1*-siRNA, and pcDNA3.1-*NORSF*. Quantitative data are plotted as mean ± standard error. ^*^P < 0.05. ^**^P < 0.01. ^***^P < 0.001

*NORSF* is a vital modulator of E2 release from sGCs [17]. Next, we investigated whether the transcription activator FoxO1 regulates E2 release by sGCs. Compared to the cells transfected with the empty vector, transfection of the *FoxO1* expression construct gave a remarkable reduction in E2 concentration in the culture medium of sGCs (Fig. 5A). In contrast, silencing of *FoxO1* remarkably increased E2 concentration (Fig. 5B), indicating that FoxO1 restrains E2 release by sGCs. Furthermore, depleting the *NORSF* transcript restrained the decrease in E2 release caused by FoxO1 (Fig. 5A), while overexpression of the *NORSF* transcript restrained the increase in E2 release caused by depleting *FoxO1* (Fig. 5B). These data suggest that *NORSF* mediates FoxO1 downregulation during E2 release from sGCs.

*NORSF* suppresses E2 release from sGCs by inhibiting the transcription of *CYP19A1*, a gene encoding an essential enzyme for estrogen synthesis [17]. Therefore investigated whether FoxO1 regulates *CYP19A1* transcription in sGCs. Transfection of the *FoxO1* expression construct gave a remarkable reduction in *CYP19A1* transcription in sGCs compared to the cells transfected with the empty vector (Fig. 5C), whereas silencing of *FoxO1* gave a remarkable induction in *CYP19A1* transcription (Fig. 5D), indicating that FoxO1 reduces *CYP19A1* transcription in sGCs. Furthermore, depleting the *NORSF* transcript restrained the decrease in *CYP19A1* transcription levels caused by FoxO1 (Fig. 5C), while overexpression of the *NORSF* transcript restrained the increase in *CYP19A1* transcription caused by depleting *FoxO1* (Fig. 5D). These data suggest that *NORSF* and *CYP19A1* axis mediates FoxO1 reduction of E2 release in sGCs.

Similarly, we also showed that, similar to its effector FoxO1, OS induces a decrease in *CYP19A1* transcription, whereas inactivation of FoxO1 can reverse this process (Fig. 5E), indicating that OS restrains *CYP19A1* transcription via FoxO1. Meanwhile, in OS triggered by H_2_O_2_ exposure, overexpression of *NORSF* restrained the increase in *CYP19A1* transcription caused by depleting *FoxO1* (Fig. 5E). These data suggest that OS restrains *CYP19A1* transcription via the FoxO1 and *NORSF* axis.

### *NORSF* is a candidate gene for sow fertility

**Fig. 6:**
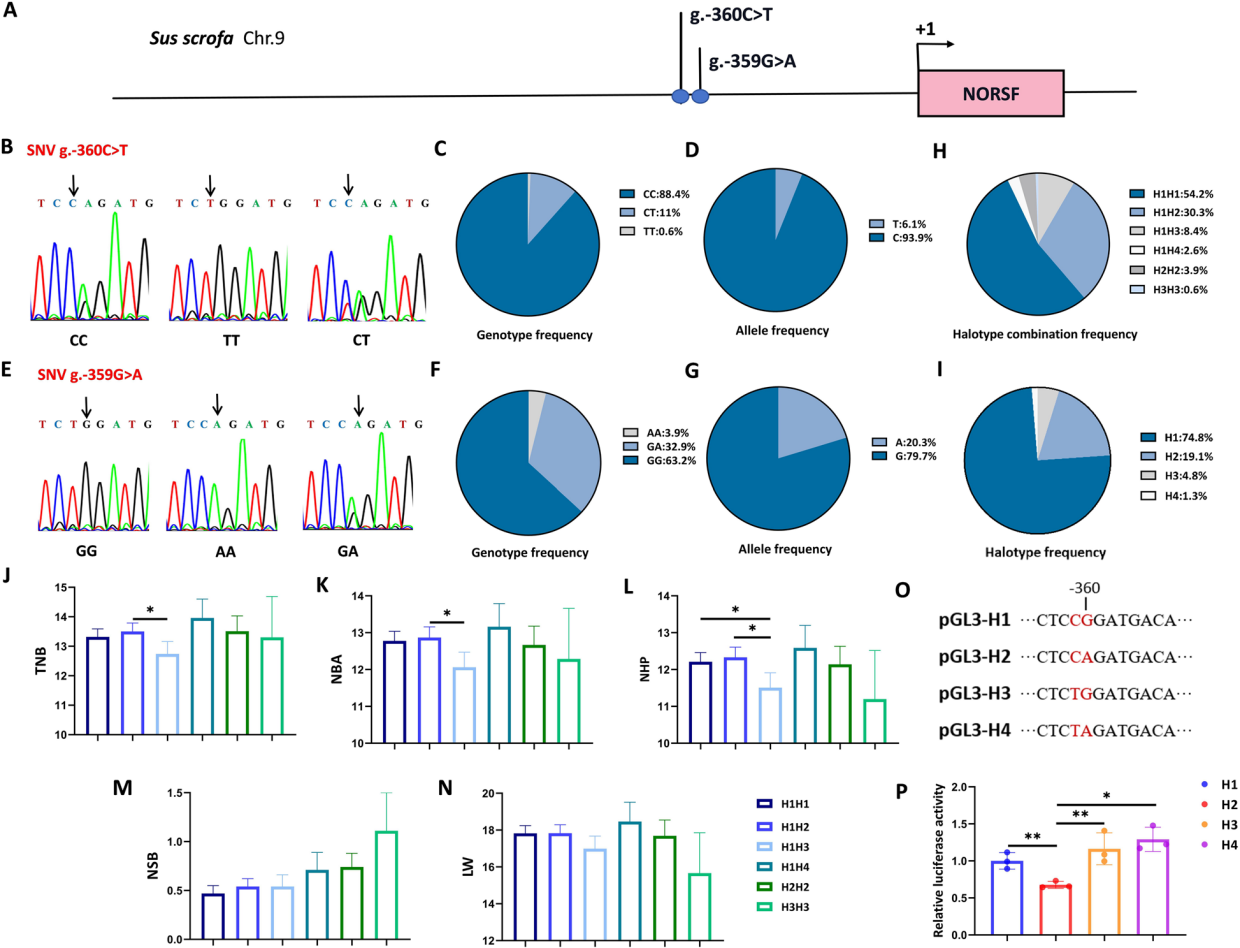
*NORSF* is a candidate gene for sow fertility traits. **A** Diagram depicting the 2 adjacent SNVs in the *NORSF* promoter. **B**–**D** Peak plot (**B**), genotype frequency (**C**), and allele frequency (**D**) of the SNV g.-360C > T. **E**–**G** Peak plot (**E**), genotype frequency (**F**), and allele frequency (**G**) of the SNV g.-359G > A. **H** and **I** The haplotype combinations frequency (**H**) and haplotype frequency (**I**) of the 2 adjacent SNVs. **J**–**N** Association analysis between the haplotype combinations and the TNB (**J**), NBA (**K**), NHP (**L**), NSB (**M**), and LW (**N**) traits. **O** Schematic showing the reporters of *NORSF* promoter with four haplotypes. **P** Reporter assay. The reporters were transfected into sGCs, and luciferase activities were measured. Quantitative data are plotted as the least squares mean ± standard error (**J**–**N**) or mean ± standard error (**P**). ^*^P < 0.05. ^**^P < 0.01

Notably, 2 adjacent SNVs (C to T and G to A) were observed at -360/-359 nt in the promoter, not the core promoter, of Yorkshire sow *NORSF* gene (Fig. 6A). In the Yorkshire sow population, three genotypes were detected for both SNVs, with the genotype AA at SNV g.-360C > T being a rare genotype with a genotype frequency of less than 1% (2/310) (Fig. 6B–G). Furthermore, these 2 SNVs were low-to-moderate polymorphic loci, with PICs of 0.108 and 0.271, respectively (Table S12). However, neither of these SNVs was significantly associated with sow fertility traits, such as TNB, NBA, and LW (Fig. S3 and S4).

Interestingly, 2 adjacent SNVs, g.-360C > T and g.-359G > A, exhibited linkage disequilibrium and formed four haplotypes: H1 (C-G), H2 (C-A), H3 (T-G), and H4 (T-A) in the Yorkshire sow population. Haplotypes H1 and H2 were major haplotypes with 74.84% and 19.03% frequency, whereas H4 was a rare genotype with a frequency of 1.29% (Fig. 6H–I). Furthermore, compared to sows with the haplotype combination H1H3, a remarkable increase in the average litter size of TNB, NBA, and NHP traits was found in sows with H1H2. The average litter size of the NHP trait was observed in sows with H1H1 (Fig. 6J–N), suggesting that *NORSF* is a candidate gene for sow fertility traits, and that H3 is an unfavorable haplotype of sow fertility.

Functional SNVs in the promoter primarily control the transcriptional activity of targets [10, 22]. To understand the impact of g.-360C > T and g.-359G > A on transcriptional activity, reporters of the *NORSF* promoter with the four haplotypes were generated (Fig. 6O). A reporter assay showed that these 2 SNVs significantly altered the activity of the *NORSF* promoter, with lower haplotype H2 activity and higher haplotype H3 activity, respectively (Fig. 6P). This is consistent with the function of *NORSF* in sGCs and the fertility phenotype of haplotype combinations. These data support that *NORSF* is a functional candidate gene for sow fertility traits, because as its 2 SNVs synergistically influence transcriptional activity and sow fertility.

## Discussion

FoxO1 is a component of the forkhead box O (FoxO) subfamily of TFs that are known to respond to many biological stressors, including OS, and play crucial roles in both physiological and disease processes [23–25]. FoxO1 serves as an effector in response to OS, with an increased activity observed in OS-stimulated cells such as sGCs [7], MCF7 cells [26], and KGN cells [27]. On the other hand, FoxO1 also acts as a TF to transmit OS signals to the nucleus, activate or inhibit the transcription of downstream genes, and mediate the regulation of cellular functions in response to OS [28, 29]. However, the extent to which the TF activity of the effector FoxO1 plays a role in OS-stimulated transcription is unknown. In this study, using lncRNAs in sGCs as an example, we showed that more than 90% of OS-stimulated lncRNAs may be transcriptionally regulated by their effector and TF FoxO1. We demonstrated that FoxO1 mediates the stimulation of the transcription of lncRNAs (e.g., *LOC100512907* and *NORSF*) in response to OS. In addition, we also noticed that multiple *cis*-target mRNAs of OS-stimulated lncRNAs have been proven to be modulated by OS [29, 30]. Interestingly, FoxO1 was the *cis*-target mRNA of OS-stimulated *LOC102160118* in *Sus scrofa* Chr.3. In contrast, as an another *cis*-target mRNA of *LOC102160118*, *SLC25A15* is an OS- and metabolism-related gene serves as a biomarker for predicting adverse events in patients with stomach adenocarcinoma [31]. 6-Pyruvoyltetrahydropterin synthase (*PTS*) and beta-carotene oxygenase 2 (*BCO2*), 2 *cis*-target mRNAs of *NORSF*, are involved in OS [30, 32]. Taken together, our findings suggest that the stimulation of lncRNome dynamics in response to OS depends on the transcription activity of the effector FoxO1.

OS and its effector FoxO1 are crucial endogenous environmental factor and TF that regulate cell state, function, metabolism, and structure [15, 33]. In the ovary, OS and its effector, FoxO1, have regulatory effects on multiple cell types, such as GC autophagic injuries, mitochondrial impairment, and apoptosis in humans, impaired viability, reduced proliferation, and cell cycle arrest in mice [34]. In addition, OS and FoxO1 also affect oocyte maturation and quality in mice, meiotic arrest in sows [35, 36], and luteal cell apoptosis and luteolysis in rats [37], and are involved in ovarian aging and diseases [35, 38]. In sGCs, OS and its effector FoxO1 are also modulators of cell viability, apoptosis, and autophagy [11, 12, 39]. OS induced by various concentrations (e.g., 100, 150, 200, 300, 500, and 1,000 µmol/L) of H_2_O_2_ and exposure times (e.g., 1.5, 2.0, 6.0, and 12.0 h) can induce sGC apoptosis and oxidative damage, whereas antioxidant factors can rescue sGC apoptosis [10–13, 40]. Furthermore, FoxO1 levels were increased in OS-stimulated sGCs and FoxO1 dysfunction rescued OS-induced sGC apoptosis, indicating that FoxO1 mediates OS-induced sGC apoptosis [13, 40]. In the present study, we demonstrated that FoxO1 mediated OS-induced sGC apoptosis. More importantly, we showed that OS restrained E2 release, a primary function of sGCs, and that FoxO1 mediated this process via *NORSF* and the estrogen synthesis pathway. Thus, our findings not only define a new function of OS and its effector FoxO1 in sGCs, but also reveal a new mechanism by which OS controls sGC functions and provides a new pathway for regulating sGC functions, the FoxO1/*NORSF*/*CYP19A1* pathway.

OS affects sow fertility by interacting with transcripts related to sow fertility [10, 41, 42]. First, multiple OS-stimulated transcripts, including mRNAs (e.g., *FZD4* and *Acvr2a*) and miRNAs (e.g., miR-192 and miR-370), are closely related to follicular atresia, which is detrimental for sow fertility [7, 11, 18, 42]. Interestingly, recent studies have demonstrated that several OS-stimulated lncRNAs such as *lnc2300* and *SDNOR* contribute to sGC function [12, 43]. In sows, downregulated *lnc2300* in atretic follicles inhibits sGC apoptosis by serving as a competing endogenous RNA of miR-365 and a *cis*-acting lncRNA of *CYP11A1* [43]. *SDNOR*, a SMAD4-dependent lncRNA, contributes to multiple sGC functions such as cell proliferation, viability, cell cycle, and apoptosis [12, 41]. Importantly, *SDNOR* also acts as an antioxidant that inhibits the induction of sGC apoptosis via OS [12]. In addition, lncRNA *NORHA* and OS can synergistically induce apoptosis by co-targeting the miR-183-96-182 cluster in sGCs [13].

Second, multiple OS-stimulated transcripts were discovered to be localized within QTLs for sow fertility traits, such as *VEGFA* (QTL ID: 584), miR-182 (QTL ID: 24290), and lncRNA *LOC100512907* (QTL ID: 7536), which are potential candidate genes for sow fertility traits. Of them, some transcripts such as *TGF-β1*, *PRLR*, and miR-27a have been demonstrated to be candidate genes for sow fertility traits [44, 45]. Several transcripts have been confirmed as the causal genes of sow fertility traits. For example, miR-23a, an miRNA located within a QTL (ID: 909) for sow fertility traits, has been identified as a causal gene for the TNB trait, because a point mutation in its promoter reduced the sGCs response to OS [10]. *NORSF*, a lncRNA activated by OS, increases during sow follicular atresia, inducing sGC apoptosis and restraining E2 release and is located in two QTLs (ID: 517 and 7462) for sow fertility traits [13, 17]. In this study, we further demonstrated that *NORSF* induced by OS is a causal gene for Yorkshire sow fertility traits (e.g., TNB, NBA, and NHP), as 2 novel SNVs in its promoter lead to transcriptional dysregulation and decreased sow fertility. However, further investigation is required to elucidate the mechanism underlying *NORSF* transcriptional dysregulation caused by these variants. Together, our findings support the conclusion that OS is deeply involved in fertility and provides a target for anti-OS therapies in sow reproductive regulation and a genetic marker (2 causal variants) for molecular breeding in swine.

## Conclusions

In summary, we provided evidence that OS stimulates the lncRNome dynamics of sGCs in an effector FoxO1-dependent manner. FoxO1 acts as a TF to interact with the FRE motifs of target promoters. Furthermore, FoxO1 mediated OS-stimulated sGC apoptosis and E2 release via the FoxO1/*NORSF*/*CYP19A1* pathway (Fig. S5). Additionally, *NORSF* induced by OS is a cause gene for Yorkshire sow fertility traits. Overall, our findings expand on the new functions of OS in female reproduction and elucidate the mechanism by which it regulates the transcription of downstream target, providing a new target for improving sow fertility through an anti-OS strategy.

## Supplementary Information

**Additional file 1: Table S1** Primers designed for reporter vector construction. **Table S2** Oligonucleotide sequences. **Table S3** Primers designed for ChIP. **Table S4** Primers designed for qPCR. **Table S5** Primers designed for genotyping. **Table S6** The putative FRE motifs in the promoters of the OS-stimulated DElncRNAs. **Table S7** *Cis*-target mRNAs of OS-stimulated DElncRNAs with the promoters containing FRE motifs. **Table S8** GO terms and KEGG pathway analysis of *cis*-target mRNAs. **Table S9** OS-stimulated *cis*-target mRNAs. **Table S10** The putative TFBSs in the *NORSF* core promoter. **Table S11** Tissue expression of the putative TFs in the NORSF core promoter. **Table S12** The polymorphism of 2 SNVs in NORSF promoter.

**Additional file 2: Fig. S1** GO analysis of *cis*-target mRNAs of OS-stimulated DElncRNAs. **Fig. S2** GO terms and KEGG pathway analysis of the putative TFs in the *NORSF* core promoter. **Fig. S3** Association analysis between SNV g.-360C > T and sow fertility traits. **Fig. S4** Association analysis between SNV g.-359G > A and sow fertility traits. **Fig. S5** Working model.

## Abbreviations

ChIP: Chromatin immunoprecipitation
DElncRNA: Differentially expressed lncRNA
E2: Estrodiol
FRE: FoxO1 response element
GEI: Gene-environment interactions
LW: Litter weight
miRNA: MicroRNA
lncRNA: Long non-coding RNA
NBA: Number of piglets born alive
NSB: Number of stillbirths
OS: Oxidative stress
sGC: Sow granulosa cell
PI: Propidium iodide
qPCR: Quantitative real-time PCR
QTLs: Quantitative trait loci
SNV: Single nucleotide variation
TFBS: Transcription factor binding site
TNB: Total number of piglets born
TF: Transcription factor
