# Supplementary material for: Oxidative stress controls lncRNA-mediated sow granulosa cell functions in a FoxO1-dependent manner
Source: J Anim Sci Biotechnol. 2024 Dec 16;15:171. doi: 10.1186/s40104-024-01120-6 (PMC11648296; doi:10.1186/s40104-024-01120-6)
Supplement: Supplementary file 2 — Additional file 2: Fig. S1 GO analysis of cis-target mRNAs of OS-stimulated DElncRNAs. Fig. S2 GO terms and KEGG pathway analysis of the putative TFs in the NORSF core promoter. Fig. S3 Association analysis between SNV g.-360C > T and sow fertility traits. Fig. S4 Association analysis between SNV g.-359G > A and sow fertility traits. Fig. S5 Working model. [file 40104_2024_1120_MOESM2_ESM.docx]

**Additional file 2**

**Fig. S1** GO analysis of *cis*-target mRNAs of OS-stimulated DElncRNAs.

**Fig. S2** GO terms and KEGG pathway analysis of the putative TFs in the *NORSF* core promoter.

**Fig. S3** Association analysis between SNV g.-360C>T and sow fertility traits.

**Fig. S4** Association analysis between SNV g.-359G>A and sow fertility traits.

**Fig. S5** Working model.


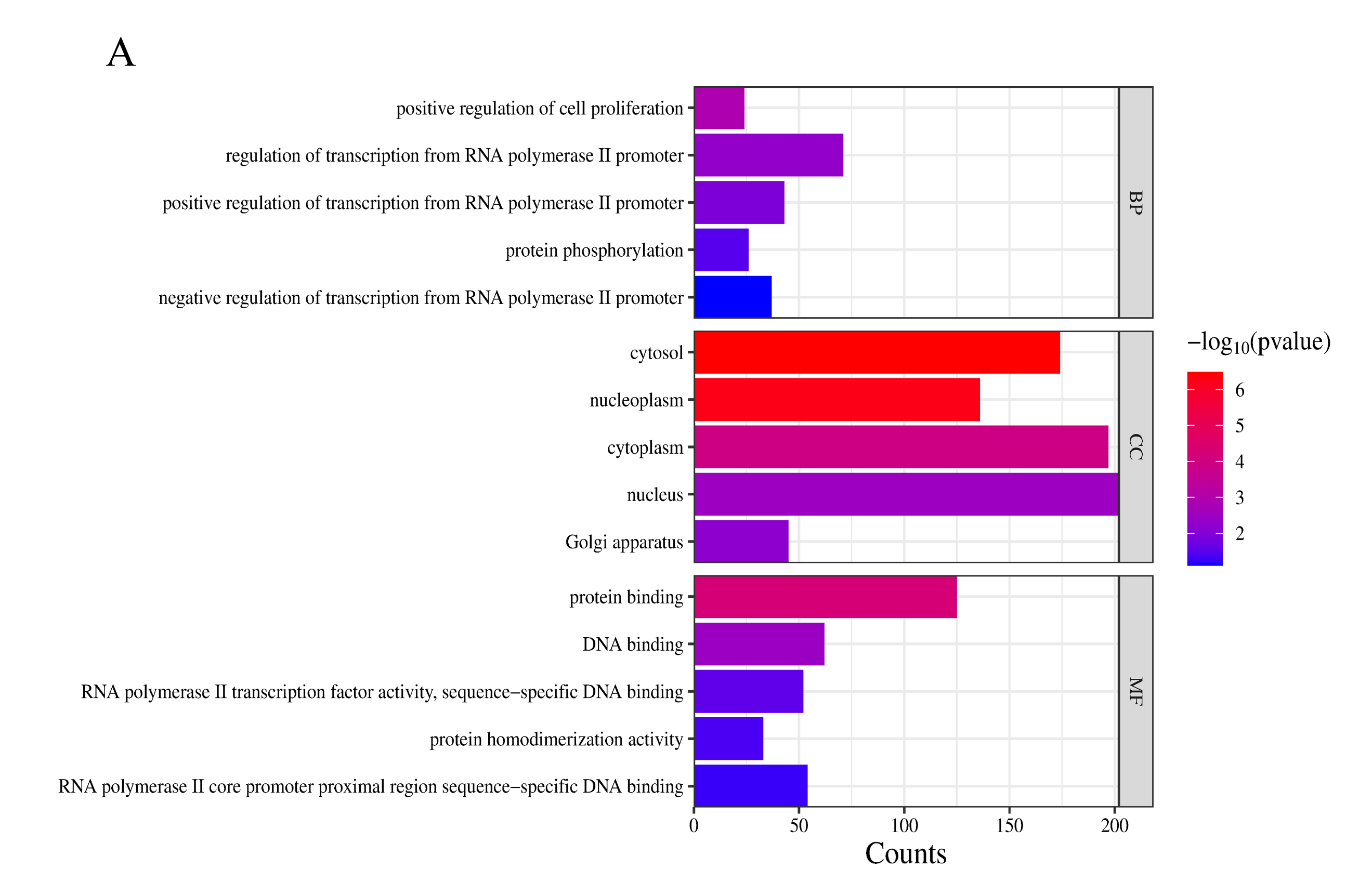


**Fig. S1** GO analysis of *cis*-target mRNAs of OS-stimulated DElncRNAs

**
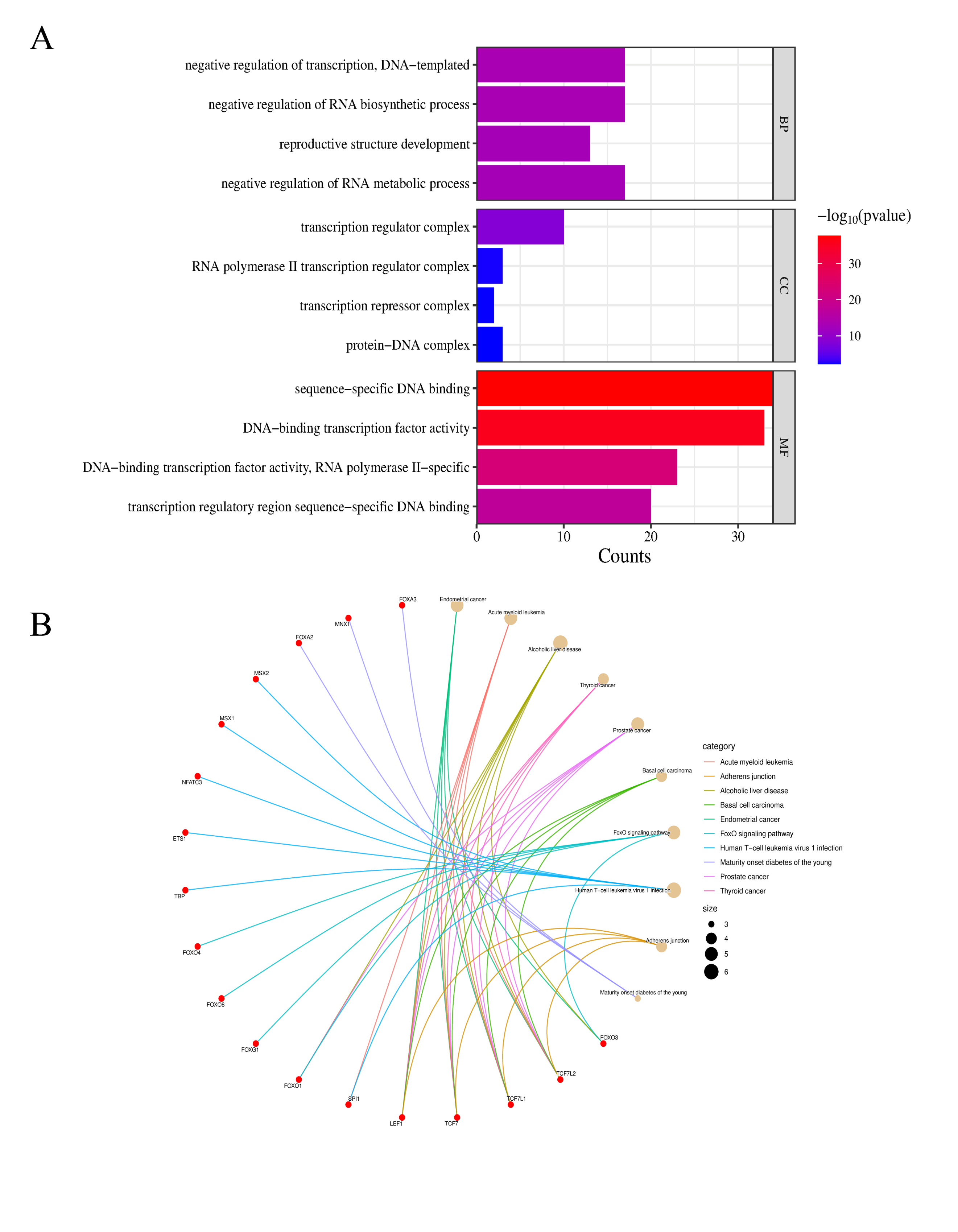
**

**Fig. S2** GO terms and KEGG pathway analysis of the putative TFs in the *NORSF* core promoter. **A** GO analysis. **B** KEGG analysis


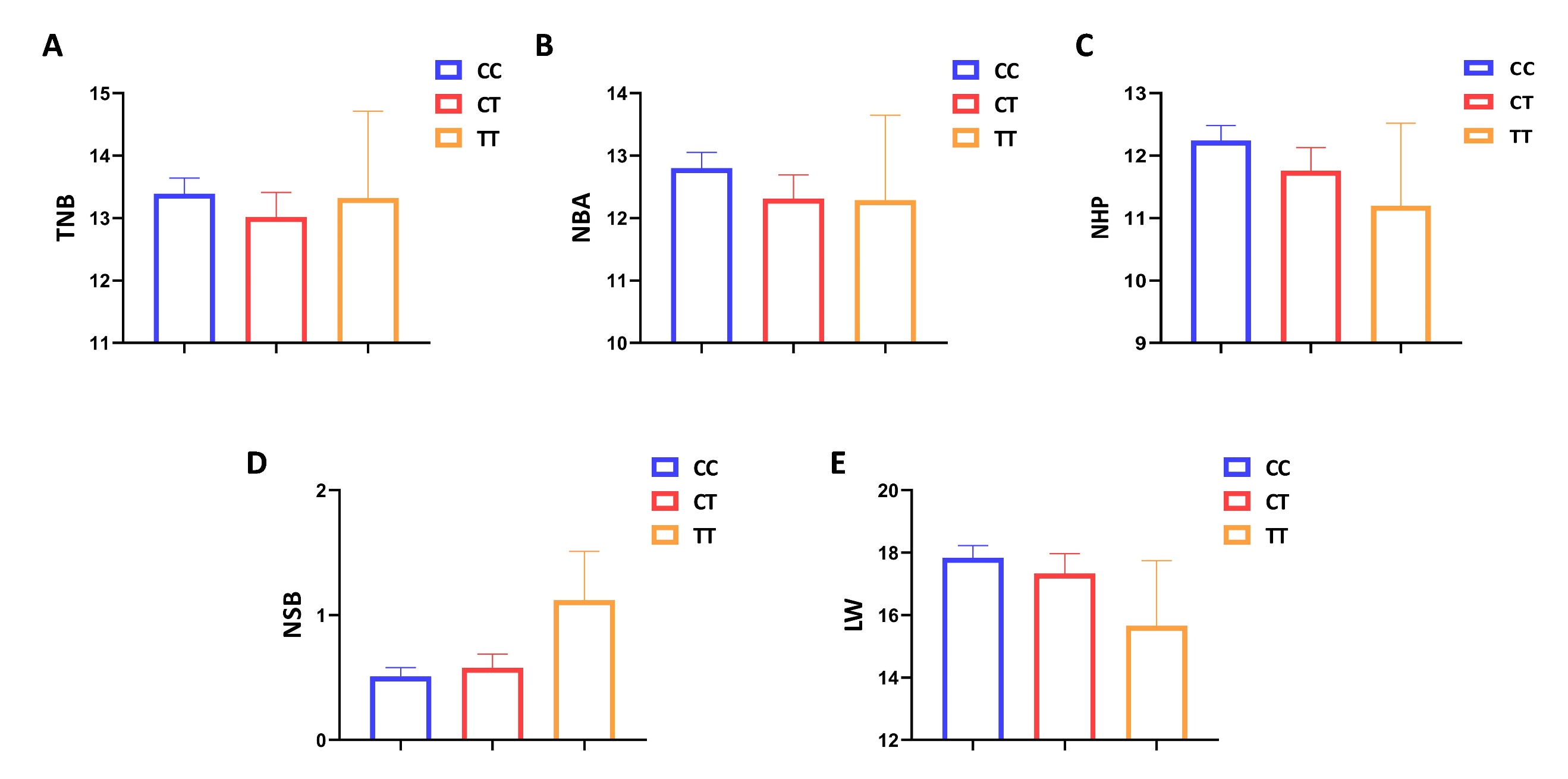


**Fig. S3** Association analysis between SNV g.-360C>T and sow fertility traits. **A** TNB trait. **B** NBA trait. **C** NHP trait. **D** NSB trait. **E** LW trait. Quantitative data are plotted as the least squares mean ± standard error


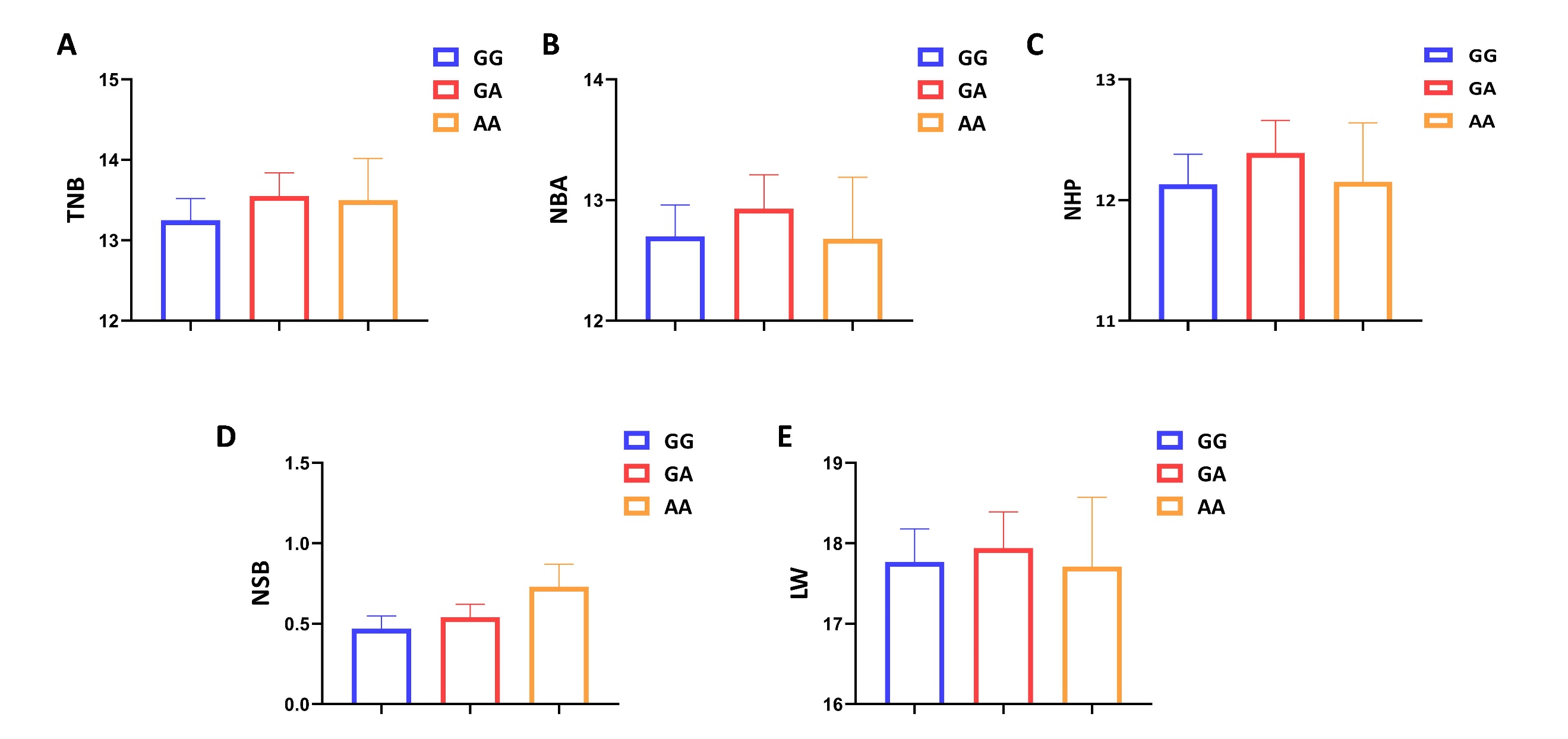


**Fig. S4** Association analysis between SNV g.-359G>A and sow fertility traits. **A** TNB trait. **B** NBA trait. **C** NHP trait. **D** NSB trait. **E** LW trait. Quantitative data are plotted as the least squares mean ± standard error.

**
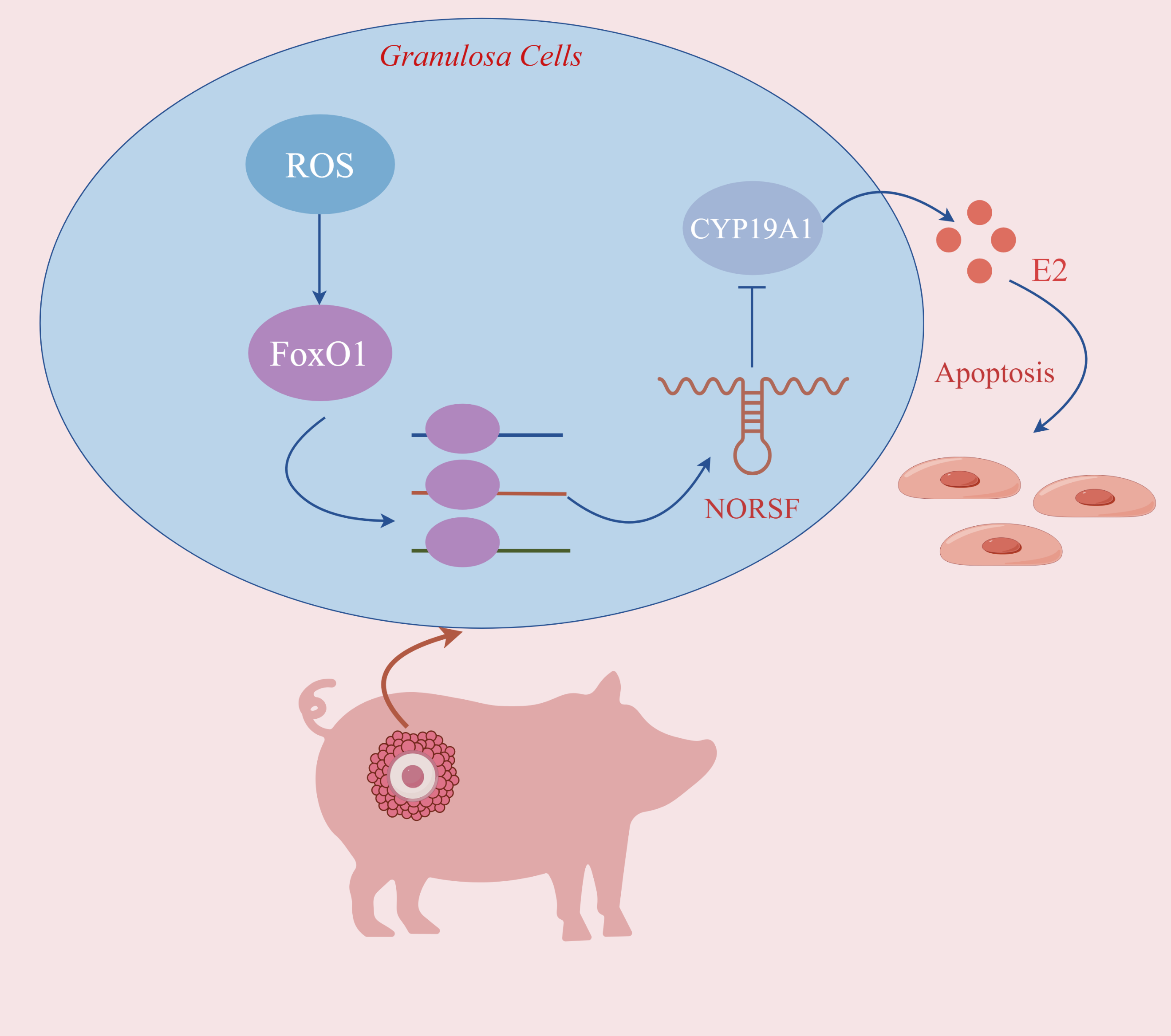
**

**Fig. S5** Working model. OS activates *NORSF* transcription via its effector and TF, FoxO1, to inhibit the expression of the *CYP19A1* gene, which encodes a rate-limiting enzyme in the E2 synthesis pathway, ultimately inhibiting E2 synthesis in sGCs
